# Uniform Tumor Spheroids on Surface-Optimized Microfluidic Biochips for Reproducible Drug Screening and Personalized Medicine

**DOI:** 10.3390/mi13040587

**Published:** 2022-04-09

**Authors:** Neda Azizipour, Rahi Avazpour, Michael H. Weber, Mohamad Sawan, Abdellah Ajji, Derek H. Rosenzweig

**Affiliations:** 1 Institut de Génie Biomédical, Polytechnique Montréal, Montréal, QC H3C 3A7, Canada; neda.azizipour@polymtl.ca (N.A.); sawan@westlake.edu.cn (M.S.); 2 Department of Chemical Engineering, Polytechnique Montréal, Montréal, QC H3C 3A7, Canada; info@recutex.ca; 3 Department of Surgery, Division of Orthopaedic Surgery, McGill University, Montréal, QC H3G 1A4, Canada; michael.weber@mcgill.ca; 4 Injury, Repair and Recovery Program, Research Institute of McGill University Health Centre, Montréal, QC H3H 2R9, Canada; 5 Polystim Neurotech Laboratory, Electrical Engineering Department, Polytechnique Montréal, Montréal, QC H3T 1J4, Canada; 6 CenBRAIN Laboratory, School of Engineering, Westlake Institute for Advanced Study, Westlake University, Hangzhou 310024, China; 7 NSERC-Industry Chair, CREPEC, Chemical Engineering Department, Polytechnique Montréal, Montréal, QC H3C 3A7, Canada

**Keywords:** spheroid-on-a-chip, spheroid, cancer, drug test, microfluidic, personalized medicine

## Abstract

Spheroids are recognized for resembling the important characteristics of natural tumors in cancer research. However, the lack of controllability of the spheroid size, form, and density in conventional spheroid culture methods reduces the reproducibility and precision of bioassay results and the assessment of drug-dose responses in spheroids. Nonetheless, the accurate prediction of cellular responses to drug compounds is crucial for developing new efficient therapeutic agents and optimizing existing therapeutic strategies for personalized medicine. We developed a surface-optimized PDMS microfluidic biochip to produce uniform and homogenous multicellular spheroids in a reproducible manner. This platform is surface optimized with 10% bovine serum albumin (BSA) to provide cell-repellent properties. Therefore, weak cell-surface interactions lead to the promotion of cell self-aggregations and the production of compact and uniform spheroids. We used a lung cancer cell line (A549), a co-culture model of lung cancer cells (A549) with (primary human osteoblasts, and patient-derived spine metastases cells (BML, bone metastasis secondary to lung). We observed that the behavior of cells cultured in three-dimensional (3D) spheroids within this biochip platform more closely reflects in vivo-like cellular responses to a chemotherapeutic drug, Doxorubicin, rather than on 24-well plates (two-dimensional (2D) model). It was also observed that the co-culture and patient-derived spheroids exhibited resistance to anti-cancer drugs more than the mono-culture spheroids. The repeatability of drug test results in this optimized platform is the hallmark of the reproducibility of uniform spheroids on a chip. This surface-optimized biochip can be a reliable platform to generate homogenous and uniform spheroids to study and monitor the tumor microenvironment and for drug screening.

## 1. Introduction

Lung cancer has been one of the leading causes of cancer death worldwide for several decades [1,2]. Notwithstanding all efforts to develop new cancer therapies, the overall survival rate of patients with lung cancer is still very low [3,4]. Most frequently, patients with advanced lung cancer develop bone metastases during the course of their disease [5]. Although these patients have a relatively short survival time, they are likely to experience debilitating bone lesions and a significant reduction in their quality of life [5,6]. One of the most challenging issues in cancer treatment is developing effective drugs in a timely manner [7]. Failure to develop novel therapies to tackle lung cancer has multiple reasons, but one of the most important reasons is the reliability of pre-clinical models to predict drug responses and their side effects in humans [7,8,9], which explains in part why drug candidates fail to achieve clinical approval more often than they succeed.

Traditionally, established cancer cell lines in two-dimensional (2D) monolayers on flat plastic substrates (e.g., Petri dishes) are used for the first phase of drug screening [7]. These culture models cannot reflect many critical characteristics of an actual tumor [7,8]. They often lack spatiotemporal chemical gradients [10] and three-dimensional (3D) cell–cell [11,12] and cell–extracellular matrix (ECM) interactions [13], which are all essential to maintain cellular functions [7] and define cell phenotypes [14,15]. 3D cell culture models have been developed over the past decades to avoid certain drawbacks of 2D culture models [7,16] by bio-mimicking the 3D microenvironment of the tumor [8,17], at least in part.

Multicellular tumor spheroids (MCTSs) stand out as the most widely used 3D cell culture models in oncology preclinical research [18,19]. MCTSs have proven their power in drug response prediction by more accurately reproducing the key structures of solid tumors [20,21,22]. However, the uniformity in size and shape of the spheroids play a critical role in their responses to anti-cancer drugs [23,24]. Therefore, it is important to produce in vitro platforms to reduce the heterogeneity of the spheroids for accurate drug testing. Various techniques have been developed for spheroid production including the hanging drop method, liquid overlay technique (LOT), spinner flasks, and stirred-tanks [7,25,26]. Advancements in microfluidics lead to the development of numerous microfluidic biochip devices to generate spheroid-on-a-chip platforms using a small liquid volume of cell suspension, as a key advantage of microfluidics [7,27,28].

Spheroids’ size, shape, and compactness level play a critical role in responses to drug compounds [24,29]. However, one of the most common problems with spheroid culture is producing spheroids with a uniform size and shape [24]. The geometric design of the channels and cell-trapping chambers play an important role in this regard [26,30,31]. However, the materials used for microfluidic fabrication and their surface characteristics (e.g., wettability, chemistry, roughness, etc.) also play a critical role in regulating cell responses to the surfaces [32,33]. Accordingly, in the past years, surface engineering has attracted great attention in cell-based biomedical applications including microfluidic cell culture platforms [34,35,36,37].

In our previous work [38], we designed a surface-optimized PDMS spheroid-on-a-chip platform to capture tumor cells and produce uniform spheroids. We used 10% bovine serum albumin (BSA) to block cell adhesion to the surface and promote cell aggregations to form homogenous spheroids. We demonstrated, for the first time, the impact of surface modification of PDMS on the uniform and homogenous spheroid on-chip production [38]. In the present study, we investigated the reproducibility of uniform spheroid formation on optimized biochips. We used the repeatability of on-chip drug testing as a hallmark of the reproducibility and homogeneity of produced spheroids. Patient-derived cells were demonstrated to retain the heterogeneity and complexity of the original tumor [34,39]. Therefore, in the present study, we investigated the capability of our surface-optimized biochip to capture tumor cells from patient biopsies and produce uniform MCTSs from patient-derived bone metastasis secondary to lung (BML) cells. Lung cancer bone metastases involve complex communications between tumor cells and the bone microenvironment [5,34]. Cross-talking between tumor cells and their surrounding microenvironment is important in cancer progression and drug resistance [11,12]. Hence, to mimic such a structure, a co-culture model of lung cancer cells (A549) and primary human osteoblasts was also developed on our optimized biochip. A 3D monotype MCTS model of lung cancer cells (A549) was produced in this study to compare with patient-derived and co-culture spheroids. We studied the efficacy of the anti-cancer drug, Doxorubicin (Dox) [40], on patient-derived MCTSs, MCTSs from lung cancer cell line A549, and a hetero-type MCTSs model (A549-primary human osteoblasts, 1:1). Here, we observed that hetero-type MCTSs and patient-derived MCTSs exhibited drug resistance to Dox more than the monotype MCTSs. In our model, we conclude that microscale interactions within sophisticated MCTSs made by patient-derived cells or hetero-type co-culture of cell lines strongly support its usefulness as a preclinical tumor model for drug screening and for studying interactions in the cancer microenvironment. To investigate the superiority of this 3D culture model in testing the therapeutic agents, we compared the drug efficacy in the 3D MCTSs models with 2D culture models using the same cells cultured on 24-well plates. We observed that drug resistance was significantly reduced in 2D culture models compared with 3D MCTSs. Our observations positively correlate with a growing body of evidence conferred by various studies [7,18,41,42], suggesting that 3D MCTSs models compared with 2D conventional culture more accurately replicate the tumor microenvironment, and therefore cellular behavior in 3D will reflect their in vivo responses more relevantly.

## 2. Materials and Methodology

### 2.1. Fabrication and Surface Treatment of Microfluidic Biochips

Microfluidic biochips were made using polydimethylsiloxane (PDMS) (Sylgard 184 PDMS elastomer kit, Dow Corning, Midland, MI, USA) as previously explained [38]. The biochips then were used for cell culture experiments seven days or more post-fabrication. Immediately before starting cell culture on-chip, channels and chambers were sterilized, and the air bubbles were removed by using 99.9% ethanol (Sigma–Aldrich, St. Louis, MO, USA). In order to decrease cell adhesion to the PDMS surfaces and increase uniformly sized spheroids, microfluidic channels were treated overnight in the incubator (37 °C, 5% CO_2_, 95% ambient air) with sterile 10% BSA (Sigma–Aldrich) solution in phosphate-buffered saline (PBS, Sigma Aldrich) as previously reported.

### 2.2. 2D Cell Culture and On-Chip Spheroid Formation

#### 2.2.1. Preparation of Cell Lines and Patient-Derived Cells

We used three types of cells for the spheroid formation and 2D culture experiments: Human lung carcinoma epithelial cell line A549 was kindly provided by the laboratory of Prof. M. Lavertu at Polytechnique Montreal. Primary human osteoblasts and patient-derived spine metastases cells secondary to lung cancer (BML) were kindly provided by Dr. M. H. Weber’s laboratory at the Research Institute of McGill University Health Centre (RI-MUHC). The ethics approval for patient sample collection was through McGill Scoliosis and Spine Group, RI MUCH REB Extracellular Matrix Protocol # 2020-5647. Resected metastatic spine tumors secondary to lung cancer were collected with consent from a patient undergoing surgery at Montreal General Hospital. By washing tissue samples in PBS (Gibco, Thermofisher) and then cutting them into 5 × 5 mm sections, samples were processed. Then, samples were incubated at 37 °C overnight in collagenase type II (Thermofisher, Gibco, Burlington, ON, Canada). Digested cells were strained using a 100 µM cell strainer and then pelleted for five minutes in a centrifuge at 300× *g*. Isolated cells consisting of a mixed population of bone metastasis-derived patient cells and bone cells were cultured in high-glucose Dulbecco’s Modified Eagle’s Medium (DMEM) (Gibco, Thermofisher), supplemented with 10% *v*/*v* fetal bovine serum (FBS) and 1% *v*/*v* penicillin/streptomycin (PS), 1% glutamax (Gibco, Thermofisher), and 1% fungizone (Gibco, Thermofisher) at 37 °C in a humidified atmosphere of 5% CO_2_.

Before processing spheroid formation on-chip, A549 cells and primary human osteoblasts (fewer than three passages) were seeded in T-75 flasks containing DMEM (Corning Inc., New York, NY, USA) supplemented with 10% *v*/*v* fetal bovine serum (FBS) and 1% *v*/*v* penicillin/streptomycin (PS) at a density of 1.5 × 10^−6^ cells per flask and were then incubated in the incubator at 37 °C, 5% CO_2_, and 95% relative humidity. When they reached 80–90% confluence, cells were washed two times with PBS (Gibco, Thermofisher) and were trypsinized with 0.25% Trypsin-EDTA solution (Gibco, Thermofisher). Then, supplemented fresh culture medium (Gibco, Thermofisher) was added to the cell suspensions followed by centrifugation for 5 min at 1500 rpm to collect the cells.

#### 2.2.2. Construction of MCTSs on the Biochips

Prior to the cell-seeding process, the channels were gently rinsed a minimum of three times with sterile PBS (Gibco, Thermofisher) to remove the BSA residues, and then the fluid within the channels was replaced with a fresh supplemented culture medium and warmed in a CO_2_ incubator. Then, the cell seeding process started by introducing a cell suspension (concentration of 1 × 10^6^ cells/mL) into the inlet of the microfluidic channel using a micropipette (P200) and removing 100 µL of the medium from the channel’s outlet. The process was repeated in the other direction by pipetting the cell suspension through the outlet and removing 100 µL from the inlet. By injecting and removing the cell suspension at the same time, we needed to make sure that channels were filled with the suspension at all times (to prevent bubble formation inside the channels). For homogenous cell distribution across the channels, this process was repeated in both directions at least three times. Microfluidic devices were put in the incubator after the cell seeding process. Culture medium was refreshed every 24 h using a P200 micropipette.

Three types of MCTSs formed on the biochip for further experiments: A mono-type MCTS model containing tumor cells A549, a co-culture of a hetero-type MCTS model containing A549 and primary human osteoblasts (1:1 ratio), and spheroids produced by patient-derived cells (BML). The cell concentration in the cell suspension was 1 × 10^6^ cells/mL in all three types of spheroids.

#### 2.2.3. 2D Cell Culture

The three types of cell suspensions mentioned above were seeded separately for the 2D culture in triplicate on 24-well plates (Falcon Tissue Culture 24 Well Plate, Thermofisher, New York, NY, USA) with the initial cell seeding of 0.03 × 10^6^ cell/well in DMEM supplemented as mentioned above for spheroids.

#### 2.2.4. Cells/Spheroids Microscopic Monitoring

Cell growth under 2D and uniformity of spheroid formation on biochip platforms were monitored daily from day one post-seeding by bright field (BF) microscopy. The culture medium was refreshed every day.

### 2.3. Drug Testing Evaluation

To assess cell sensitivity to anti-cancer drugs, doxorubicin (Dox, Sigma–Aldrich) was chosen as an anti-cancer drug model. Dox was stored at 1000 mM aliquots in PBS at −20 °C and diluted to the desired concentrations in PBS. Immediately before the drug test experiments, three concentrations (0.1 mM, 1 mM, and 10 mM) of Dox in low-serum media (1% FBS) were prepared. As a control group, 0 mM of Dox in media was prepared using PBS.

Three days after cell seeding in the microfluidic biochip, compact and uniformly sized spheroids were treated by either sterile PBS vehicle or drug (Dox) under low-serum conditions (1% FBS) for two days. Culture medium containing the drug or PBS was introduced into the channel inlets and placed in the incubator for 24 h. The medium was exchanged after 24 h with a freshly prepared medium containing the relevant dose of drugs or PBS and incubated for another 24 h (a total of 48 h of drug treatment).

To compare differences and similarities in 2D and 3D culture responses to Dox treatments, we compared their sensitivities to Dox. For this purpose, monolayer 2D cell cultures were treated with either the sterile PBS vehicle or Dox 48 h after cell seeding in 24-well plates for a duration of two days. The media containing the drugs or PBS were refreshed every day during the two days of drug treatment. For each concentration of Dox, at least three independent experiments were performed for both 2D and 3D models.

#### 2.3.1. Metabolic Activity and Proliferation Assay Post-Drug Treatment

To assess 2D and 3D cell viability after exposure to the selected concentration of drugs for 48 h, Alamar Blue (Thermofisher) assay was carried out according to the manufacturer’s instructions. Briefly, after removal of the drugs from the cells/spheroids, Alamar Blue dye was added to the culture media at 10% vol on the last day of drug treatment (48 h after drug exposure) and incubated with cells/spheroids in the incubator (37 °C, 5% CO_2_) for 4 h. After incubation, the fluorescence intensity at excitation wavelength 560 nm and emission wavelength 590 nm was recorded using a Tecan Infinite M200 Pro microplate reader (Tecan Trading, AG, Männedorf, Switzerland). The metabolic activity was presented as a percentage of the negative control (cells/spheroids untreated with Dox) from the same series of experiments. Three independent experiments (with three replicates each time) were performed for each cell type and each concentration of Dox. After the measurements, spheroids were washed with PBS for further fluorescence-based live/dead assay.

#### 2.3.2. Differential Staining

To visualize the distribution of the live and dead cells in the spheroids on the last day of the experiments, a fluorescence-based live/dead assay was performed using the Live/dead Viability/Cytotoxicity kit (Invitrogen, Sigma–Aldrich) according to the manufacturer’s instruction. Two days after drug exposure and immediately after the Alamar Blue assay, spheroids were washed with PBS and incubated with the live/dead staining solution for 20 min protected from light in a 37 °C incubator. An Epifluorescence inverted microscope (Axio Observer.Z1, Zeiss, Oberkochen, Germany), sCMOS camera (LaVision, Göttingen, Germany), and the objective lens EC Plan-Neofluar with respective fluorescence filter for Calcein AM (ex 485 nm, em 530 nm) and ethidium bromide EthD-1 (ex530 nm, em 645 nm) were used to capture spheroid images.

### 2.4. Statistical Analysis

All experiments were repeated for three independent replicates (n = 3) in order to evaluate the reproducibility of the data. For each experiment, a minimum of 10 spheroids were considered for analysis at each time point per condition. Statistical analyses were performed by using Microsoft Excel 2016 (v1803, Build 9126.2259). All data are represented as the mean ± standard error of the mean. We performed one-way ANOVA to assess statistical significance between means, with post-hoc Tukey tests for comparison between means. A *p* ≤ 0.05 level was considered statistically significant.

## 3. Results and Discussion

Cells randomly aggregate to form spheroids in conventional spheroid culture methods (e.g., 384-well plate) [31]. As a result, the spheroid size and shape can be non-homogenous, which leads to the difference in diffusion distance of the drug within various spheroids [31,40]. However, to accurately evaluate the drug efficacy on spheroids, the generation of uniform-sized, compact, and spherical spheroids is essential for comparison of the responses under various drug concentrations [23,24,29].

### 3.1. Biochip Fabrication and Surface Treatment

**Figure 1:**
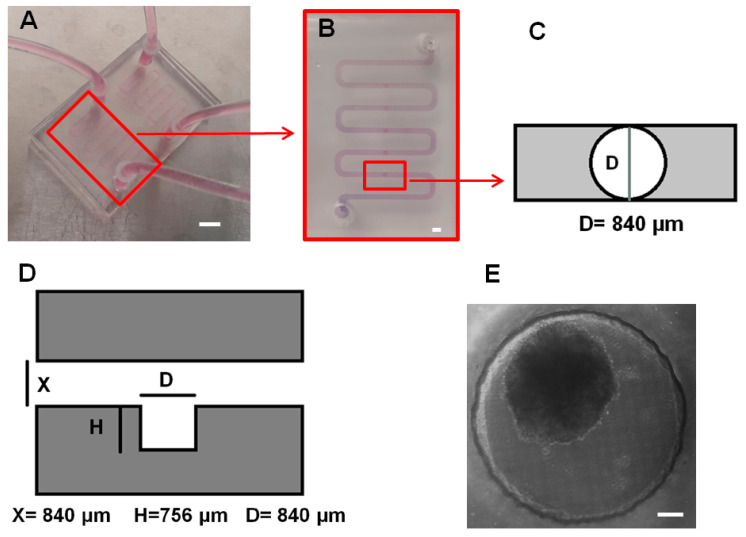
Microfluidic biochip design and spheroid formed in a cylindrical cell-trapping chamber. (**A**) Image of two layers of the PDMS microfluidic device fabricated by the soft lithography technique. Each device has two independent channels of 109.2 mm in length with a cross section of 840 × 840 μm. Each channel has five cylindrical cell-trapping chambers for cell sedimentation and spheroid formation. The error bar represents 1 cm. (**B**) Top view image of a single channel with inlet, outlet, and five cylindrical cell-trapping chambers. The error bar represents 1 mm. (**C**) Schematic of the top view of the cylindrical cell sedimentation trap. The diameter is 840 μm. (**D**) Side view schematic of the cell-trapping chamber. The diameter of the cylindrical chamber is 840 μm, and the height of the chamber is 756 μm. X represents the size of the cross-section of the channel, which is 840 μm. (**E**) Microscopic image from day five of a lung tumor (A549) spheroid formed in the cell-trapping chamber. The scale bar represents 100 μm.

Microfluidic platforms, by providing relatively uniform spheroids compared to conventional spheroid formation methods, were demonstrated to be a reliable in vitro platform for testing drug efficacy [21,27,31]. We developed an optimized microfluidic biochip surface treated with 10% BSA, which was statistically demonstrated to be successful to form uniform and compact spheroids compared to non-treated devices [38]. This optimized surface treatment, by decreasing the cell-surface interactions [24], promotes tumor cells to self-aggregate more tightly and produces compact and homogenous spheroids. We used stereolithography to fabricate the resin mold, and consequently soft lithography was performed as explained before for the fabrication of PDMS biochips. Each device has two layers of PDMS that are permanently bonded with oxygen plasma. Figure 1 represents the images of the biochip, microfluidic channel, and an image of a lung tumor A549 spheroid captured inside the cylindrical chamber by a bright-field microscope.

### 3.2. Cell Culture and Spheroid Formation Experiments

#### 3.2.1. Spheroid Formation on Biochips and Drug Test

**Figure 2:**
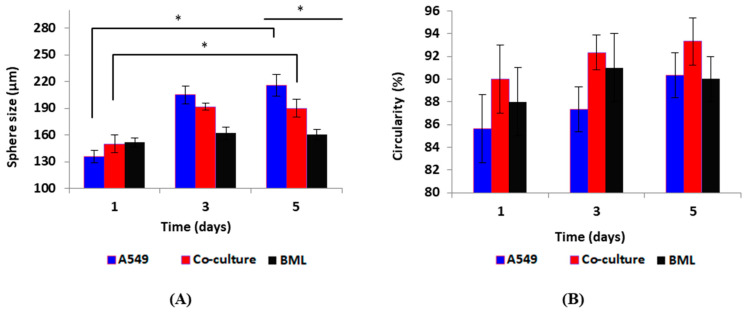
Spheroids size and circularity during the five days of culture. (**A**) Spheroid size changes in three groups during the five days of culture. Spheroid size increased significantly on day 5 compared to day 1 for mono-culture and co-culture spheroids (D1 vs. D7 * *p* < 0.05). The increase in the size of BML spheroids was not statistically significant when comparing day 5 to day 1. On day 5, the size of the three groups of spheroids was significantly different when compared to each other (* *p* < 0.05). (**B**) Change in circularity in the spheroids during the five days of culture. This shows how the spheroids’ shape is spherical and uniform. Spheroids in all three groups had a homogenous circularity and spherical form. Circularity did not change statistically significant when comparing day 5 to day 1 for the three groups. Circularity was not significantly different on day 5 when comparing the three groups. The total initial number of cells was 1 × 10^6^ cells/mL in all three groups. *p* < 0.05 was considered statistically significant. All assays were performed as triplicates per trial in three independent experiments. (Error bars represent ±SE, n = 3).

In this work, we produced a mono-type MCTSs (A549) (named group A), a hetero-type MCTS (A540: primary human osteoblasts, ratio 1:1) (named group B), and a patient-derived MCTS model (BML) (name as group C). Our main goal was to investigate the capability of this optimized biochip to produce uniform and homogenous spheroids on-chip. The chemosensitivity of the anti-cancer drug, Doxorubicin (Dox), was studied as a hallmark of the reproducibility and uniformity of the spheroids. Prior to investigating the drug sensitivities of these three cell conditions, the ability of these cells to form uniform-sized and compact MCTSs on our surface-optimized biochip was assessed in accordance with our previous work [38]. Figure 2 demonstrates changes in the size and circularity of the spheroids. We used the same method as our previous work to calculate circularity. Circularity is defined as the percentage of the minimum diameter (Dmin) divided by the maximum diameter (Dmax) around a single spheroid. This parameter shows if spheroids are uniformly shaped and spherical. By qualitatively monitoring the three groups of cells (A, B, C), we observed that mono-culture and co-culture spheroids demonstrated significant increases in size on day 5 when compared with day 1 (*p* < 0.05). The size of BML spheroids did not significantly increase from day 1 to day 5. This difference can be explained by the fact that primary cells mostly grow slower than the cell lines. Moreover, in spheroids made by primary cells, more dynamic cellular interactions and strong adhesion within the tumor microenvironment are expected to play a role to form tight cell–cell connections. This could be some of the reasons for no marked changes in their size when comparing day 5 to the first day of culture. Since the PDMS layers are transparent, the size and shape of each spheroid can be easily observed using an optical microscope without the need for fluorescence cell labelling before spheroid formation.

**Figure 3:**
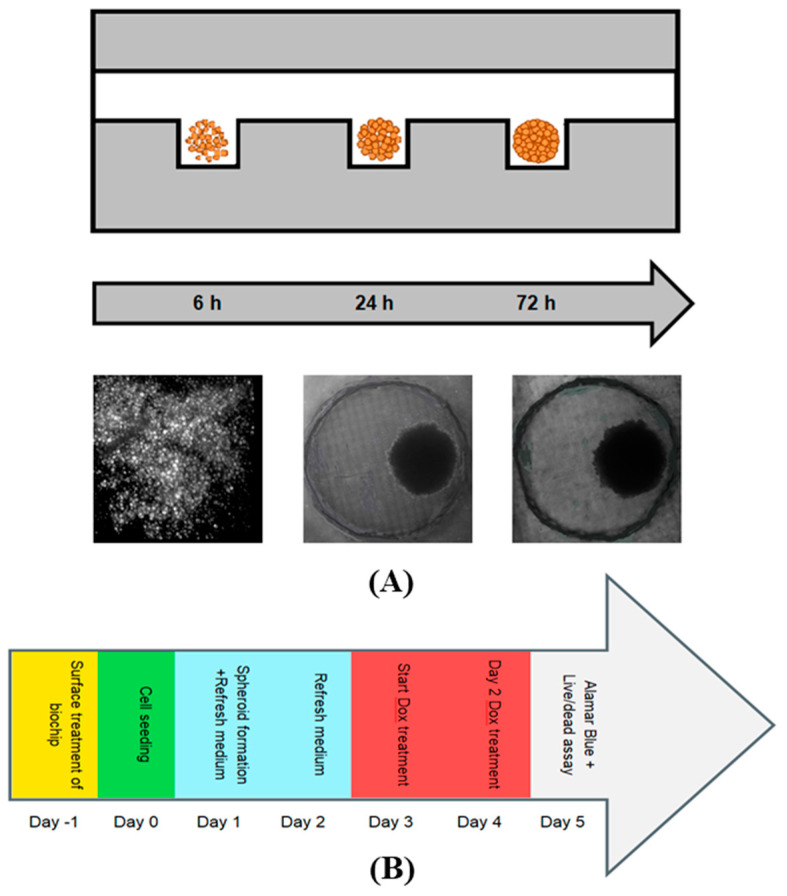
Schematic and images of the cell sedimentation process and spheroid formation in the microfluidic cell-trapping chamber and the experimental timeline for drug tests. (**A**) The cell suspension is introduced through the channel’s inlet, and some of the cells will settle down into the cell-trapping chambers due to gravity. As the PDMS surface of the channel is treated with 10% BSA and is cell repellent, cells effectively agglomerate and form compact and uniform spheroids on day three after cell seeding. At the bottom of the figure, we see images of A549 lung cancer cells that produced spheroids captured by bright-field microscopy. (**B**) Experimental timeline for the spheroid formation and drug test on the biochip. The start of the drug dosing was on day 3 post-seeding, and on day 5 post-seeding (after 48 h of drug exposure), drug response analysis was performed.

Spheroid formation on-chip has been achieved due to gravity by sedimentation and containment [43,44]. As the PDMS surfaces are pre-coated with 10% BSA, the cell-repellent properties of the surface [45] and the cylindrical shape of each cell-trapping micro-well enabled the precise and quick cell agglomerations. Due to the adhesive interactions between cell-surface proteins and the extracellular matrix, the spheroids were formed [24,46]. The cell aggregation of A549, co-culture of A549: primary human osteoblasts (1:1), and the spine metastasis patient-derived cells (BML) was observed 24 h after the cell culture on-chip. However, the compact spheroid formation was observed three days after the cell seeding (Figure 3A).

The drug screening on-chip was performed according to this timeline: the day of the cell seeding was considered day 0, and 24 h after the cell seeding was considered day 1. On day 3, the spheroids are more compact, spherical, and larger in size when compared with day 1. Therefore, drug administration was performed on day 3 for 48 h. Then, on day 5, drugs were removed from the devices and the Alamar Blue assay was performed followed by live/dead viability staining on the same day. We monitored spheroid growth, and the culture medium was exchanged on a daily basis. For the two days of drug treatment, the culture medium with the corresponding concentration of the drug was refreshed once a day. Figure 3B represents the timeline of the experiments.

#### 3.2.2. 2D Culture Model as Compared with 3D MCTSs

In the drug development process, having appropriate in vitro pre-clinical tools provides a major contribution to more precisely predicting drug responses in clinical trials. To assess drug sensitivity in 3D MCTSs compared with the 2D model, we compared our optimized microfluidic platform with a 2D monolayer culture model on 24-well plates for all three cell conditions.

Here, we evaluated Doxorubicin (Dox) sensitivity in three groups of cells: mono-type A549 cells, hetero-type A549: primary human osteoblasts, and patient-derived cells using both 2D (24-well plate) and 3D (MCTSs) in vitro models. Doxorubicin [40] is known as one of the most widely used chemotherapy agents in the early and advanced treatment of several types of cancer. However, tumor resistance has limited the effectiveness of Dox in single-drug treatment [31]. The mechanism of drug resistance is poorly understood [40].

### 3.3. Drug Testing Evaluation

#### 3.3.1. Metabolic Activity Testing Post-Drug Treatment Using the Alamar Blue Assay

To assess the metabolic activity after drug exposure, the Alamar Blue assay is performed due to nontoxicity of Alamar Blue to cells [47]. Forty-eight hours after drug administration, culture media were removed from the microfluidic channels and 24-well plates and replaced with Alamar Blue solution, which was incubated with spheroids in microfluidic channels and with the cells in 24-well plates to measure the metabolic activity of the cells/spheroids. Alamar Blue assay [41] defines the viability of the cells/spheroids and the toxicity effect of Dox on cells. Resazurin [47] is an oxidation–reduction (REDOX) indicator of Alamar Blue that undergoes colorimetric change (from blue to red) when reduced to fluorescent Resorufin by metabolically active cells. Resorufin can be reduced to non-fluorescent hydroresorufin later. Cells and spheroids untreated with the drug (0 mM Dox) were considered the control group. Cell/spheroid viability after drug treatment was calculated in relation to the control group. A blank sample was prepared using only dye solution (10%vol Alamar Blue). The incubation time with Alamar Blue was adjusted experimentally by incubation of 10%vol Alamar Blue with cells/spheroids at 37 °C for 1, 2, 3, 4, 5, 6 h. After each time, the fluorescence intensity was recorded; 4 h of incubation was selected for all experiments as the highest fluorescence intensity signal was detected after 4 h of incubation. Fluorescence intensity was measured using fluorescence spectrophotometry and red fluorescence while excitation and emission wavelengths were set to 560 nm and 590 nm, respectively.

All groups demonstrated a decrease in metabolic activity compared to the control when the drug concentration increased. However, this decrease was not statistically significant except for mono-type spheroids A549 whose metabolic activity significantly decreased compared to the control when exposed to 1 μM and 10 μM of Dox (1 μM Dox vs. control * *p* < 0.05 and 10 μM Dox vs. control * *p* < 0.05). Spheroid co-culture and patient-derived spheroids exhibited more resistance to Dox administration compared to the mono-culture. This drug resistance could be related to tumor–stromal cell interactions that are considered crucial factors of cancer cell growth and their resistance to anti-cancer therapies [48,49]. The interactions between the tumor cells and their microenvironment modulate cellular signaling, which has an effect on tumor cell responses to chemotherapeutic agents [49]. Our results demonstrate that the co-culture models and using patient cells can better reflect the in vivo-like tumor conditions in vitro.

**Figure 4:**
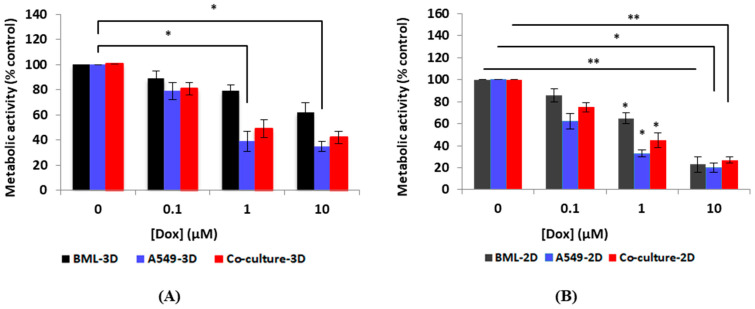
Metabolic activity changes compared to the control group for three groups of cells in 3D MCTSs and the 2D model. (**A**) Metabolic activity of MCTSs measured after 48 h of drug exposure. All three groups of spheroids demonstrated a decrease in metabolic activity compared to the control under increased drug concentration. However, the decrease in cellular metabolic activity was not statistically significant in other groups except for mono-type spheroids A549; the decrease in metabolic activity was significant compared to the control when exposed to 1 μM and 10 μM of Dox (1 μM Dox vs. control * *p* < 0.05 and 10 μM Dox vs. control * *p* < 0.05). MCTSs from patient-derived cells demonstrated more resistance to Dox compared with the other two groups. (**B**) Metabolic activity measured in the 2D model (24-well plates). All three groups of cells showed a decrease in metabolic activity with increased drug concentration. The decrease in metabolic activity was non-significant when cells were exposed to 0.1 μM Dox; however, metabolic activity significantly decreased compared to the control when the cells were exposed to 1 μM and 10 μM of Dox (1 μM Dox vs. control * *p* < 0.05 and 10 μM Dox vs. control * *p* < 0.05 and ** *p* < 0.001). *p* < 0.05 was considered statistically significant. All assays were performed as triplicates per trial in three independent experiments. (Error bars represent ±SE, n = 3).

The Alamar Blue assay results showed when the cells were exposed to Dox in the 3D model, they demonstrated greater resistance than the cells in the monolayer under all three cell conditions. Most of the spheroids in all groups treated with Dox maintained a spherical shape, but many of the spheroids under high drug concentrations displayed a rough surface and could be considered dead. A similar trend was observed in all three groups. In all three groups, spheroids treated with all concentrations of Dox (0.1, 1, and 10 mM Dox) showed lower metabolic activity compared with the spheroids in the control group (0 mM Dox) (Figure 4). In the 2D model, all three groups of cells showed a decrease in metabolic activity with increased drug concentration. This decrease was non-significant when cells were exposed to 0.1 μM Dox. However, metabolic activity significantly decreased compared to the control when the cells were exposed to 1 μM and 10 μM of Dox (1 μM Dox vs. control * *p* < 0.05 and 10 μM Dox vs. control * *p* < 0.05 and ** *p* < 0.001) in all groups (Figure 4).

At the high concentration of Dox (10 mM), most of the spheroids became loose and lost their compact and circular shape, which can be related to general cell death. Spheroids subjected to lower concentrations of Dox (0.1 μM, and 1 μM) maintained their integrity, but their growth was limited and therefore the detected fluorescent signal was lower compared with the control group.

**Figure 5:**
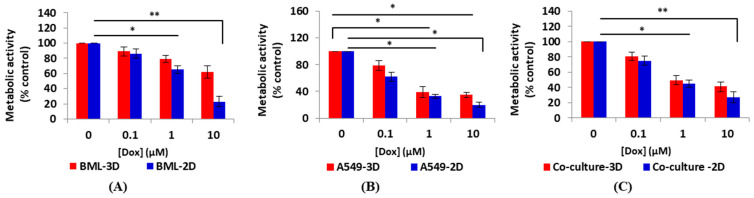
Comparison of metabolic activity reduction after drug exposure in 3D and 2D culture for each group. In all groups, cells were more resistant to drugs in 3D MCTSs. (**A**) Patient-derived cell metabolic activity in 3D MCTSs and 2D model. Spheroids are drug resistant as drug penetration is more difficult in 3D form. Therefore, the metabolic activity of spheroids decreased non-significantly compared to the control group when they were exposed to various concentrations of drugs. However, a decrease in metabolic activity was observed by increasing the drug concentration, but this decrease was not statistically significant. Cells in 2D demonstrated statistically significant decreases compared to the control group when exposed to 1 μM (* *p* < 0.05) and 10 μM (** *p* < 0.001) of Dox. (**B**) Established lung cancer cells A549 showed a significant decrease in metabolic activity when exposed to 1 μM of Dox and higher under both 3D and 2D conditions (* *p* < 0.05). (**C**) Co-culture of lung cancer cells A549 and primary human osteoblasts at a ratio of 1:1 to mimic the tumor–stromal reaction. Metabolic activity of spheroids decreased non-significantly when compared to the control group by increasing the Dox concentration. In the 2D model, metabolic activity decreased statistically significantly compared to the control when the cells were exposed to 1μM (* *p* < 0.05) and 10 μM (** *p* < 0.001) of Dox. *p* < 0.05 was considered statistically significant. All assays were performed as triplicates per trial in three independent experiments. (Error bars represent ±SE, n = 3).

We observed that treatment of cells with Dox in the 2D culture for two days inhibited cell proliferation and survival dramatically. Compared with the 2D models, MCTSs in all three groups had greater resistance to Dox and better demonstrated in vivo-like interactions between drugs and cancer tissues. The slower proliferation rate of cells grown under the 3D conditions compared to the 2D monolayer culture plays an important role in drug resistance since cytotoxicity may be less effective in cells with a slower proliferation rate when compared to cells with a higher growth rate. When cells grow in 2D, the cells spread in a monolayer on the culture surface. Therefore, when a drug is tested in 2D, the drug components need to only diffuse the distance across the cells to reach its target. However, in 3D, cells are arranged in multilayers and the drug needs to diffuse across layers of cells to reach its target. This could be one explanation for higher drug resistance in 3D MCTSs compared with the 2D model. Figure 5 shows the comparison of metabolic activity between 3D MCTSs and 2D for each group of cells.

Our results demonstrate that our surface-optimized biochips, by providing a cell-repellent surface, produce homogenous MCTSs that can serve as a reliable in vitro tool for drug sensitivity assays. These observations were supported by live/dead assay images presented in the following section.

#### 3.3.2. Differential Staining (Live/Dead Assay)

**Figure 6:**
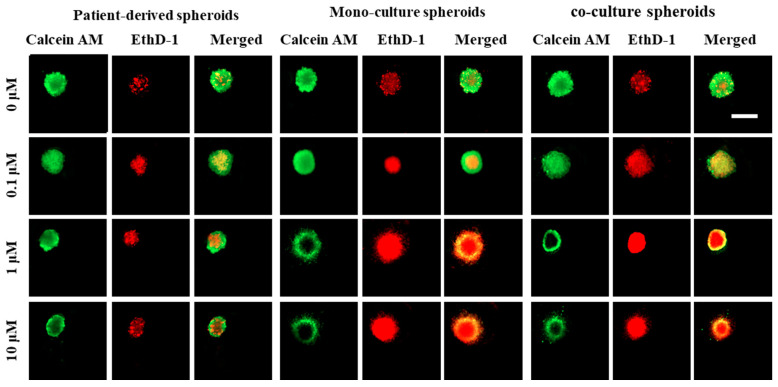
Images of differential staining (live/dead assay) captured by fluorescent microscopy. The green color is due to the Calcein AM and it represents the distribution of the live cells in the spheroids. The red color is due to the EthD-1 and it represents the dead cells in the spheroids. In all three groups of cells, an increase in the area of dead cells and a decrease in the area of the live cells were observed by increasing the drug concentrations. The initial total number of cells was 1 × 10^6^ cells/mL for all experiments. A ratio of 1:1 was considered for the co-culture model of lung tumor cells A549 and primary human osteoblasts by keeping the total initial cells at 1 × 10^6^ cells/mL. All assays were performed as triplicates per trial in three independent experiments. Images represent a repetitive phenomenon observed in three independent experiments. The error bar represents 200 μM.

On the last day of the experiments, differential staining and microscopic observation were performed to observe the live and dead cell distributions in the spheroids. As these compounds are toxic to cells, this experiment was carried out as the last additional reference method to confirm the results obtained in the Alamar Blue assay. A mixture of PBS containing Calcein AM and ethidium bromide EthD-1 according to the manufacturer’s guideline was introduced into the microfluidic channels and incubated at 37 °C for 20 min. After this time, spheroid images were captured using a fluorescence microscope. Living cells (green fluorescence) and dead cells (red fluorescence) were observed in the recorded images. Figure 6 shows how the number of live (green color) and dead (red color) cells changed for spheroids exposed to different concentrations of Dox compared to the control group in all three groups of cells.

Changes in the cellular viability and spheroid morphology can be associated with cell death, as apoptotic and necrotic cells lose their ability to stay tightly connected to other cells. The microscopic observation of the live/dead cell viability assay showed that spheroids exposed to a higher concentration of Dox demonstrated irregular edges, which can represent un-aggregated necrotic cells in the outer layer of the spheroids that were affected by the high concentration of the drug. However, the creation of a necrotic core inside the spheroids, which is due to the gradient of nutrients, oxygen, and harmful metabolites in the center of the spheroids, can release inter-cellular death signals that can influence the cell viability in all the layers of the spheroids.

One of the challenges in using the live/dead assay in the 2D culture compared to the 3D model is that the dead cells usually detach from the surface in monolayer (2D) culture plates. Therefore, they are easily removed during the pipetting of the live/dead solutions. Hence, the results demonstrate higher viability compared to the actual viability due to the under-representation of the dead cells in the monolayer culture. Therefore, for 2D models, other methods (e.g., flow cytometry) are more appropriate to determine live/dead cell populations when we first remove all the cells (live and dead) from the culture plates and then stain and image/count them to reduce the under-counting effect of dead cells. Accordingly, in this work, we only used the live/dead staining method as a complementary technique for the Alamar Blue assay for spheroid models only, as the main objective of this work is to assess the repeatability of these surface-optimized biochips to produce uniform spheroids for reliable drug tests.

The co-culture and patient-derived spheroids had higher sensitivity to Dox. These spheroids are more compact (dense) due to the stronger cellular interactions inside the spheroids [29]. There is some evidence that the increase in the hypoxic center inside the spheroids has a positive correlation with the compactness level of the spheroids [50,51]. Hypoxia is known to be one of the important factors in drug resistance in spheroids [51]. Hence, we can conclude that hypoxic status in patient-derived and co-culture spheroids can be one of the causes of their resistance to chemotherapeutic drugs. The phenomenon of spheroids showing more drug resistance compared with monolayer culture is not well known and has been attributed to various mechanisms. These can include decreased drug penetration into the cells, hypoxia inside the spheroids, and up-regulation of genes imparting drug resistance.

From our results, we can conclude that mono-type MCTSs cannot accurately recapitulate the in vivo-like tumor microenvironment. As tumor cells’ interactions with stromal cells play a critical role in mimicking the tumor microenvironment in vitro, co-culture models of tumor cells with stromal or patient-derived 3D MCTS models might better represent the tumor features in vitro.

MCTSs represent the in vivo-like features of the tumor microenvironment with respect to gradient distribution of oxygen, nutrients, metabolites, and drug penetration. Therefore, having the other key components of tumor tissues (e.g., immune cells, fibroblasts, osteoblasts, etc.) will increase the in vivo-like characteristics of MCTS models. We assume that MCTSs can significantly contribute to the development of optimized cancer treatment for patients. Our results showed the potential of our surface-optimized biochips for reproducible production of uniform and homogenous spheroids toward reliable and repeatable drug testing.

## 4. Conclusions

The fact that numerous anticancer drugs are eliminated during the clinical phase of drug development demonstrates that current 2D culture models are not efficiently predictive. The 3D tumor spheroids recapitulated the feature of the tumor microenvironment more precisely and better estimated the efficacies of the chemotherapeutic drug compounds compared to the 2D models. However, one of the most important challenges in using spheroids for drug testing is producing uniform spheroids [23,24,29].

In the present work, we developed surface-optimized microfluidic biochips for uniformly sized MCTS formation, culture, and drug testing. Uniform spheroids from lung cancer cell line A549, co-culture of lung cell line A549 and primary human osteoblasts, and patient-derived spine metastasis secondary to lung (BML) cells were produced in these optimized platforms in a repeatable manner. The modified PDMS surfaces of the biochips provided cell-repellent properties. Therefore, cell–cell interactions became dominant over cell–surface interactions. This led to the reproducible formation of compact and uniform spheroids on-chip. We observed that drug test results were repeatable, which is a hallmark of reproducibility. 

Overall, the present study highlights the importance and suitability of our in vitro model for drug testing. In conclusion, these surface-optimized microfluidic platforms may be convenient in vitro tools for uniformly sized and homogenous spheroid formation and culture from established tumor cell lines, co-culture models, and patient-derived cells. Further surface optimization of these biochips is the subject of work in progress to better elucidate the efficacies of these platforms and to serve as a reliable drug-testing devices for developing new therapeutic agents and for optimizing the existing drug compounds in personalized medicine.

## Data Availability

Not applicable.

## Abbreviations

BBB	blood–brain barrier
BSA	Bovine serum albumin
DMEM	Dulbecco’s Modified Eagle’s Medium
ECM	Extra cellular matrix
EthD-1	Ethidium homodimer-1
MCTS	Multi cellular tumor spheroid
PBS	Phosphate buffered saline
PDMS	Polydimethylsiloxane
PVA	Polyvinyl alcohol
SD	Standard deviation
2D	2 dimensions
3D	3 dimensions
